# A Treatise on Diseases of the Urethra

**Published:** 1833-07-01

**Authors:** 


A Treatise on the Urethra, and its Diseases, especially Stricture, and their Cure. By *Benjamin Phillips*, Author of a Series of Experiments made to demonstrate that Arteries may be obliterated without Ligature, Compression, or the Knife. 8vo. pp. 319, two Plates, London, 1832.

If we may judge from recent publications, the attention of surgeons is at present directed a good deal to the subject of stricture. Mr. Stafford has written on the division of stricture, Mr. Brodie has transformed his Lectures on the Diseases of the Urinary Organs into an octavo volume, and we have this brochure of Mr. Phillips before us. Our readers will see as we go on, whether our present author has anything peculiar to offer, and without further preface we shall plunge at once *in medias res*, and select what portions appear most fitting to develop Mr. Phillips' sentiments, or explain his practice.

The first chapter treats of the Anatomy of the Urethra. The only passage that we think it worth while to notice, is that relating to the direction of the canal, which our author, as will be seen, has endeavoured to ascertain.

"The words used by some authors of the present day, 'that the canal is straight, or almost straight,' having left much uncertainty in the minds of practitioners, I have determined here to endeavour to shew the exact direction of the canal, confident as I am that the knowledge will be found of much utility in practice. On six subjects, examined under ordinary circumstances, and in a healthy state, the rectum and bladder being carefully emptied of their contents, the level of the most depending part of the prostatic region of the urethra was found to vary from three to five lines below the level of the most depending portion of the vesical orifice of this canal. The level of the most depending portion of the membranous region averaged nearly nine lines below the most depending portion of the vesical orifice,—and the distance between the vesical orifice and the point of the triangular ligament through which the urethra passes was fourteen lines—that point being three lines inferior to the vesical orifice of the canal. From this admeasurement, it must be evident that, in the state of health, the last curvature is inconsiderable.

On two subjects, having hypertrophy of the prostate, which I had an opportunity of examining, though the bladder was much depressed in the pelvis, the level of the most depending portion of the prostatic region of the urethra was found from six to seven lines and a half below the level of the internal orifice of this canal. There can be no doubt that like variations would be frequently found if new and extended researches were made on the subject; but these observations alone are sufficient to shew, that, in sounding, it is necessary at this point to elevate more or less the beak of the sound.

In children, the canal undoubtedly presents a greater curvature; for, in infants, the small capacity of the pelvis does not admit of the descent of the organs which at a later period it is destined to contain. As its development increases, these organs are more completely contained in the cavity of the pelvis, and the canal has then a tendency to approach to a straight form. One circumstance which tends to shew that the urethra is or may be made nearly straight without difficulty, is, that little pain is experienced by the introduction into the canal of a straight instrument: indeed, we find that even when curved instruments are employed, frequently the whole of the curved portion has been passed into the bladder—and that all that remains in the canal is straight." 20.

Certainly it is not difficult, under ordinary circumstances, to introduce a straight instrument into the bladder of either the living or the dead subject. The plan which we have found answer best is this: elongate the penis well upon the instrument and pass it steadily on till its point hitches in the bulb; then withdraw it for about half an inch or an inch, and again pass it forwards, taking care to keep the point elevated, when the sinus of the bulb is avoided, and the instrument enters the membranous part of the canal. The second chapter is devoted to the pathology of the Mucous Membranes, and it appears to us to treat a little too much de omnibus rebus. Neither is our author particularly clear on some occasions, as the following sentences will shew.

"By inflammation I understand that succession of phenomena occurring in a tissue, and produced by an irritation, of whatever kind it may be. They are super-excitation of the vascular system, succeeded by an enlargement of the vessels, and a certain stagnation of blood in their cavities. This congestion or semi-stagnation is produced as soon as the circulation (at first accelerated) begins to relent. Once produced, it may occur, that, by the force of impulsion which serves to produce it, the capillaries may be ruptured: there is then extravasation. At other times, this fluid is deposited in the substance of the tissue by a true exhalation; so that there may be, according to these two modes, extravasation of blood, or serum, fibrine, or pus. This infiltration, which is affected in the interstices of the tissue, adds to the irritation produced by the vascular congestion; it increases the sensibility, and produces a tensive pain.

This state, so complicated in appearance, termed inflammation, I reduce then to three morbid modifications—vascular super-excitation, vascular congestion, and interstitial infiltration. These are its elements, and they are narrowly connected the one with the other. This state is characterized principally by a redness; by an increase of temperature; by a tumefaction; and by an augmentation of the sensibility of the tissue. Redness, heat, pain, and swelling, such are the characters of inflammation." 31.

We must confess that the foregoing elimination of our author's ideas on Inflammation is not of the most satisfactory description, and he assumes rather more than a cautious reasoner would venture to do. For instance, it is not absolutely certain that in inflammation, super-excitation of the vascular system is succeeded by an enlargement of the vessels, and a certain stagnation of blood in their cavities. Neither do we see very clearly how a force of impulsion would produce stagnation, on the contrary we should rather conclude that, cæteris paribus, any increase of impulsion would rather tend to prevent it, unless there were some obstruction to impede the current so impelled. We also think that our author deals rather too freely in general assertions, or rather enunciations of general principles or laws. He says, for example, that the pain accompanying inflammation of the mucous system, *never* acquires that intensity which it does in similar affections of cellular, serous, fibrous, or even osseous tissues. It is most true that inflammation of mucous membranes is not usually characterised by acute pain, and had Mr. Phillips contented himself with stating the general fact, none could have disputed it. But when he says that inflammation of mucous membranes never produce severe pain he evidently goes too far. There are few tortures greater than those excited by the presence of stone in an inflamed bladder, or by ulceration of the mucous membrane in the prostatic part of the urethra. We might attack several positions of Mr. Phillips, which seem to us to be far from impregnable, though very confidently held, but we will not occupy our readers' time with criticisms of this description.

Our author, after adverting to the pseudo-membranes formed by the mucous membrane in croup, &c. observes, with reference to the genito-urinary system—

"Although we have very little evidence to shew that pseudo-membranes are produced in the genito-urinary system, it is not probable that system is exempt from the affection. I am aware of only very few cases which establish the existence of pseudo-membranes in the genito-urinary system: one is cited in the *Journal Géneral de Medecine*, vol. 68, page 206, and was transmitted by M. Destrees, a physician at Vailly, to the Society of Medicine at Paris. In that case the individual who was affected with cystitis and intestinal inflammation expelled by stool a portion of false membrane, so extended as to alarm him, believing he had voided his intestines; he at the same time passed his urine with much pain, and with it a thick mucus mixed with considerable portions of membraniform concretions or pseudo-membrane." 41.

Mr. Phillips next describes the pathological conditions and effects of chronic inflammation. He notices the vegetations or excrescences sometimes, though rarely, observed. They appear to be a deposition from small vessels, and are surrounded by a tissue at first reddish and gelatiniform, but which becomes greyish, dense, and indurated, in proportion to the period of the continuance of the inflammation. Mr. Phillips directs attention to the small number of recorded instances, in which the effects of chronic inflammation of the urethra have been ascertained by inspection after death. The following is a brief summary of our author's opinion as to those effects, and their influence in occasioning contractions of the canal.

"I have now passed in review a certain number of changes accompanying or succeeding to inflammation, and which have a tendency to contract to a greater or less extent the diameter of the canal of the urethra.

The first is that change which occurs during the progress of acute inflammation of the mucous membrane, in which tumefaction or engorgement exists to so considerable an extent as to cause occasional retention of urine.

The second is that state of thickening of the mucous membrane alone, or accompanied by a similar affection of the submucous cellular tissue, which generally occurs as a consequence of chronic inflammation in the canal.

The third is produced by those vegetations, succeeding to a chronic inflammation of this tissue, which project into and necessarily lessen the diameter of the canal.

The fourth is that state of the canal where the inflammation has terminated in an adhesion of its parietes, and in which a tolerably distinct septum, or valvular replication, is occasionally found. The mucous membrane, at the situation of these valves, appears reeved almost as if a thread were ran through its thickness and then tightly drawn. Morgagni describes the progress of the last change in the following manner: certain erosions of the membrane are replaced frequently by some slight excrescences, which, in contracting, present at first fibres, and these become more and more dense, and form at last white lines which project in the cavity. Laennec supposed these 'excrescences' to be false membranes, formed by a plastic exudation and supported sometimes by a large and prominent base. If, however, what I have already advanced with regard to pseudo-membranes be correct, this opinion can have no foundation." 50.

Mr. Phillips concludes this chapter by some reflections on the reproduction of mucous membrane. He cites Andral, Cruveilhier, &c. in support of the declaration that they are reproduced, and he instances the analogy of the membranes lining fistulous canals. The only appreciable difference between new and normal mucous membranes, is said to be the absence of follicles in the former.

The third chapter is dedicated to urethritis. There is little in it to which we could profitably direct attention, the whole being rather a compilation of the jarring opinions of other men, than an exposition of original experiments or original ideas on the part of Mr. Phillips. We shall merely allude to a few insulated points. Those who have seen much of venereal practice, must have occasionally been consulted by patients who complain only of pain in the course of the urethra. As Mr. Phillips observes, this most frequently succeeds inflammation accompanied with discharge. We have two patients under our care at the present time affected in this manner. The pain is sometimes severe, aggravated on the passage of the urine, and attended with gleety discharge. This affection is rare, usually of long duration, and proves a source of great annoyance to the patient. Mr. Phillips relates the following case, which is not devoid of practical interest.

"I have seen only one case of this affection. A man of 28, in good health, who had never before suffered from any affection of the sexual organs, had, a year previous to my seeing him, an urethritis, which was discovered on the tenth day after connexion, and which, having existed eight days, terminated spontaneously. From that period he had experienced in the urethra extremely acute pain, unaccompanied by discharge. Rigid diet and a freedom from exercise of a fatiguing kind caused the disease to disappear; but when his exercise became fatiguing, or his diet irregular, the pain returned with violence, especially during the emission of urine or semen. His general health continued good; and in some months after the discharge had disappeared, he had connexion with two or three females, to whom he did not communicate the affection. The penis presented no appreciable alteration; a sound of large size was admitted without difficulty; the jet of urine was as large as usual. He had been directed to rub, on the inferior surface of the penis along the canal of the urethra, mercurial ointment, in combination with camphor, extract of belladonna, and hydriodate of potash: opium and mercury were administered internally without success. After much anxious reflection, we determined to produce a discharge from the urethra by introducing a bougie smeared with virus, obtained from a patient suffering from contagious urethritis. It was however deemed prudent to endeavour first to produce a discharge by simply irritating the canal: accordingly, an irritating injection was introduced into the urethra. The injection produced a profuse discharge, which terminated in a few days, and with it the pain which had produced so much anxiety and distress." 86.

In the patients under our own care, to whom we have already alluded, we have employed counter-irritation, applied to the inferior surface of the penis, combined with the use of alkaline aperients and opium. We must own that, hitherto, we have derived little benefit from these means. In both these patients there is gleety discharge, and we feel strong doubts as to the propriety of Mr. Phillips' idea of re-inoculating the patient with gonorrhœa. The pain is probably kept up by some source of irritation in the urethra, and we should imagine, à priori, that steady persistance in counter-irritation, and the avoidance of all causes likely to aggravate the complaint, would prove the best plan of treatment in the majority of cases. But such notions being rather conjectural than actually supported by decisive facts, we would not attach more importance to them than they merit.

Mr. Phillips, in discussing the treatment of that very obstinate affection, chronic urethral discharge, or gleet, adverts to M. Lallemand's employment of the nitrate of silver in substance. The following short case will illustrate this statement.

"M. A. retained, during a long period, a chronic discharge from the urethra, experiencing at the same time, as a constant symptom, pain and uneasiness in the fossa navicularis. He was treated, during a year, with injections, the composition of which was varied; without deriving any benefit from their administration. The surgeon under whose care he had placed himself applied the nitrate of silver eighteen or twenty times to the fossa navicularis, without obtaining any alteration of the symptoms. Pasquier was consulted, and felt assured of the existence of an ulceration under the symphisis of the pubis. He made a single application of the nitrate of silver to this portion of the canal, and in eight days the discharge had entirely ceased." 109.

It will be observed that the application of caustic is not limited to the anterior portion of the canal, and it cannot be denied that in long-continued urethral discharges the posterior portion is affected. Whether the direct application of lunar caustic is likely to succeed much better than the long train of injections, bougies, cubebs, copaiba, lytta, turpentine, and the many other drugs and applications that have been and daily are directed against gleet, we will not pretend to determine. We cannot but fear that this mode of treatment will more frequently than the others induce inflammation of the bladder or the testicle, those pleasant corollaries to gonorrhœa and the remedies employed for it. M. Lallemand's statement of the effects of the application of caustic are not very explicit.

"The inflammation produced by cauterization may be inefficient; it will then be necessary to repeat the application. It may be too energetic; and should then be moderated by ordinary means. Lallemand says that, far from seeking to dissimulate with regard to the incoveniences of this remedy, he would endeavour to exaggerate them, in order that they may be avoided by the suggestion of the necessary precautions. He states that, nine times in ten, he has cured, by cauterization, discharges of long standing, which have resisted treatment the most rational and varied. There exists this difference between cauterization and other remedies—that its success is more durable, because it acts directly on the diseased tissue, and changes its organization." 114.

But we must quit the subject of gonorrhœa; and we confess that our author's remarks upon it are neither calculated to render our notions on the subject more definite, nor our practice more systematic. The next chapter is on catheterism. Some of our readers may not be aware of the antiquity of instruments for exploring the urethra. Troja and Lassus state that the sound with two curvatures, the invention of which has been referred to Petit, had been in use two thousand years before his time by the Greek surgeons. Lassus had seen in the Museum of Portici, near Naples, this kind of sound, which had been discovered in the ruins of Pompeii; it, as well as all the surgical instruments of the Greeks, was of bronze; it had the same form, the same length, and the same diameter as that of Petit, and differed only in this, that, in place of two lateral holes, near its extremity, it had only one, which was on the concave portion of the sound, and near the extremity. Paulus Equieta, who lived in the early portion of the seventh century, in speaking of diseases of the urethra and of the treatment they require, says—that when ulcerations exist in the urethra, we should introduce into the canal a quill, or a tent made of linen, and covered with some desiccative ointment. The following remarks on the introduction of instruments along the urethra into the bladder are practically very good.

"In introducing an instrument into the urethra or bladder, it is necessary to recollect that the two opposed parietes of the urethra—the superior and inferior—differ singularly as to their configuration, and that we cannot indifferently follow the one or the other with the beak of an instrument. The inferior portion is yielding; for neither along the penis, nor at the height of the scrotum, nor beneath the pubic symphisis, is it supported by any thing solid. In gliding along the canal, the beak of the sound may easily enough push before it the lining membrane of the urethra; for along its surface we meet, in old men, inflections of the membrane (resulting from its flaccidity), which occasionally have a tendency to arrest the progress of the instrument. Those inflections are, however, principally longitudinal, and they cannot arrest the passage of an instrument. Some orifices of mucous follicles, and among others those of the glands of Cowper, are, according to general opinion, susceptible of receiving and arresting the beak of a sound, especially if it be of a small size. Lastly, at the level of the bulb and in front of the contour of the neck of the bladder, on the sides of the verumontanum, there exists on the inferior surface marked depressions, the orifices of which are presented towards the external orifice of the urethra; against these the beak of the sound passes, and is by them occasionally prevented from making further progress.

If we examine, by means of dissections (attentively made) false passages formed in the urethra, during life, or after death, in subjects submitted to catheterism, we find that they are produced by the rupture of the inferior parietes of this canal, and that the greater number of those passages exist either at the situation of the depression I have pointed out or at the bulb. The disposition of the superior part of the urethra is infinitely more favourable as a conductor for instruments in their passage along the canal than the inferior. Sustained in front by the corpora cavernosa, and behind by the pubic symphisis, it presents great firmness; and we find only longitudinal replications, which are removed by the distension produced by the instrument; and no obstacle is here presented to its progress: no depression exists here under ordinary circumstances; there is no projection of the prostrate into this portion of the tube; neither do we find many follicles, either isolated or grouped; nor any considerable orifice. I limit myself here to these succinct indications concerning the anatomical disposition of the urethra, having entered largely into detail when describing the anatomy of this organ." 130.

It is impossible for any man to introduce instruments safely and successfully, unless he keep the point of the instrument directed towards the superior rather than the inferior wall of the urethra; and it is owing to ignorance or neglect of this precaution, that we see catheters or sounds passed in the bungling and the mischievous manner in which they frequently are. The following quotation will shew that straight instruments have no better claim than have curved to be entitled a modern invention.

"The use of straight instruments is not new in surgery; for they have been discovered in the ruins of Herculaneum and Pompeii. Khalaf-Ebn-Abbas-Abu'l-Kasem, generally known by the name of Albucasis, who died in 1122, and who wrote a work on the operations of surgery, which is one of the most precious relics of the age in which he lived, gives a representation of a straight instrument; but there are no directions for its use. But it is evident that they were in use in his time; and as there exists so much doubt as to Albucasis having written another work, in which allusion is made to this instrument, we may still more confidently support the opinion. Friend has, I think, proved that the work which has commonly been attributed to Alzaharavius, who is supposed to be a different person from Albucasis, was only a part of a great work of the latter. Lieutaud, who lived in the middle of the eighteenth century, expresses himself thus:—'But we may avoid this operation (puncture of the bladder), always dangerous, and often fruitless, because it does not remove the cause of the disease, in providing ourselves with a straight sound, solid or hollowed. I can assert, from the knowledge I have of these parts, healthy or diseased, that there is not any case, if we exempt that of stone existing in this canal, which can prevent a straight sound, conducted by a hand a little practised, from entering into the bladder.' Since that period, they have been used by men of distinguished character, and have acquired an ephemeral reputation." 138.

Our author's cautions against the employment of force in the introduction of instruments, whether straight or curved, are judicious. We now pass to the second part of the work, which is more particularly devoted to the consideration of stricture. The first chapter of this part is on the definition, origin, classification, situation, and symptoms of stricture. Those who are curious to learn our author's ideas on this subject must consult the work itself. We do not see anything peculiar or striking in them. The only passage to which we shall refer is one upon the seat of stricture. In a hundred and seventy-three cases which our author has selected, the disease was situated at the following distances from the orifice of the urethra. In nine the distance did not exceed an inch, in eight from one to two inches, in thirteen from two to three inches, in eleven from three to four inches, in ninety-eight from four to five inches and a half, in forty from five and a half to six and a half inches, in ten from six inches and a half to seven and a half. Mr. Phillips justly remarks, that there is a considerable difference in the length of the anterior part of the canal in different individuals, and that, although the distance from the orifice may vary, the seat of the stricture may remain the same or nearly so. Mr. P. too thinks that in the cases alluded to above, the disease, when at a greater distance from the orifice than four inches and a half, was seated either in the neighbourhood of the curvature of the urethra, or between that point and the prostatic portion of the canal, and that the difference in the admeasurement was dependent on the length of the organ.

The second chapter contains a history of the treatment of stricture. Our author observes that there are two leading principles of treatment commonly acted on—dilatation of the strictured canal, and destruction of the stricture.

The latter is ordinarily effected by cauterization, but incision is sometimes required. We are almost tempted to extract a historical notice of bougies and escharotics, as it may amuse those who take an interest in observing the progressive improvement of all useful inventions. We must, however, pass on to the consideration of the treatment. After stating that bougies have the effect of dilating, compressing, and irritating the canal, Mr. Phillips proceeds to urge the following objections to the treatment by dilatation.

"When most exempt from accident, the treatment by bougie is long, occupying three, six, nine, or even twelve months, and requires great precaution on the part of the practitioner and much patience and resignation on the part of the sufferer. During the time occupied in the employment of the bougie, the patient is frequently required to keep the instrument in the urethra through the night, and sometimes day and night; he is obliged to withdraw the bougie whenever he wishes to urine, and afterwards to reintroduce it; and in many cases he is prohibited from taking the slightest exercise. The necessity of confinement and a repose more or less absolute, is a serious inconvenience, especially if the patient be old, feeble, or irritable; and the presence of the bougie produces frequent interruption to, if not total loss of, sleep. The necessity of removing the bougie to urine is also in some cases an inconvenience of the highest magnitude, because much difficulty is frequently experienced in the reintroduction of the instrument. The treatment by bougies, too, implies the possibility of passing the instrument through the stricture, which is not always practicable; for, after placing the bougie in contact with the stricture and using gentle pressure, we are unable to pass it, either from the orifice of the stricture not being in the axis of the canal, or, if situated in the centre of the canal, being so very narrow, that the bougie is unable to penetrate the obstruction. It frequently occurs that, after having passed through one obstacle, we meet a second, a third, or a fourth, which successively interrupts the introduction of the instrument, and, should it finally obstruct its passage, renders any progress which may have been made ineffectual. After having succeeded in introducing a bougie into the bladder, it not unfrequently happens, when we wish the next day to repeat the introduction, that we find, from the irritation of the previous operation, we are unable to effect our object." 181.

Mr. P. remarks in addition that the bougie is frequently bent back upon itself—that sometimes the irritability of the urethra is so great that the patient cannot support its presence in the canal for five minutes—that if it be allowed to remain for a night, or even for a much less period of time in that portion of the canal which remains healthy, it may give rise to inflammation and stricture—that if there be more than one obstacle and the bougie has dilated the first, it is necessary to employ a smaller bougie for the next, when the former stricture has a tendency to relapse—and, finally, that in the greater number of cases there is a recurrence of the disease. Mr. Phillips observes in conclusion,—

"If to these observations I add, what I assume has been already proved, that it is in the early stages of the disease only, we can hope (even with all the precautions which have been recommended) to succeed by the use of bougies, the reader will be astonished that dilatation is so generally employed, and will expect the exclusive advocates of that treatment to cling with less pertinacity to their favourite remedy." 188.

And further on he adds,—

"I have now passed in review the methods of treating strictures by bougies and by sounds, and have, I submit, established their inapplicability in the vast majority of cases. I have shewn, that by far the greater number of strictures are an effect of induration existing in the parietes of the canal—that this induration can only be removed by dilating bodies, through the medium of absorption —that, if induration has long existed, absorption can no longer be performed—and that bougies or sounds can therefore succeed in the earlier stages of the disease only; whilst it is notorious, that application for medical assistance is rarely made until the disease has acquired a formidable character. Absorption being, then, available for the cure of strictures in a very early stage of the disease only, and compression being, as I apprehend, wholly inefficient for the removal of the obstruction, there remains but one mode by which, in the case of organic stricture, we can restore the canal to its original capacity, and that mode is destruction of the stricture; and this is effected by means of a caustic substance or a cutting instrument." 193.

We have no hesitation in saying, and we think most practical surgeons will agree with us, that the foregoing is a most exaggerated picture of the difficulties, and a most imperfect and unjust estimate of the success of the treatment by dilatation. So far from thinking it inapplicable in the vast majority of cases, we know, from experience, that it is applicable in that vast majority; and this statement convinces us that Mr. Phillips is no very practical man. We have seen and treated many cases of stricture; we have witnessed the treatment of very many at our hospitals; and in very few instances indeed has it been impossible to introduce an instrument into the bladder. It is true that this was not always done at the first attempt, but by gentle perseverance it was effected in almost every instance. An instrument being introduced, those difficulties that form so alarming a picture in Mr. Phillips' pages have never presented themselves to or been observed by us. By a gradual increase of size in the instrument the stricture was steadily and effectually dilated, without risk, and comparatively speaking, with little pain. Mr. Phillips observes that the disease is not permanently cured. It certainly is not; but where is the great inconvenience of a patient's introducing an instrument for himself, once in the week or the fortnight? We do not find that patients regard this necessity as one of intolerable hardship. But in point of fact this tendency to relapse is observed after the cauterizing and incising plan of treatment, as well as after that by dilatation. Whatever the advocates of the caustic, or the stilette may assert, the public may be assured that this is the truth. And after all, when their modes of procedure are scrutinized, it will be found that they really employ dilatation in their procedures, and affect the chief good by its means. The third chapter is on the employment of caustic. The gist of all our author's reasonings and arguments is to shew that caustic was not and could not be successful as formerly employed by Hunter and Home, because it was merely directed against the anterior surface of the stricture, but that when directed, as he recommends it to be, from within outwards, the objections vanish or are much diminished, and the benefits are vastly increased. We shall not describe Mr. Phillips' mode of applying the caustic, as in fact he gives no systematic description of his manipulations; but we may state that he first employs what he calls his model bougie to take an exact cast of the stricture, and then introduces the caustic by means of a canula or *porte-caustic*. We are induced to waive any further attempt at description, because in the number of this Journal for

July 1832, we gave a very full account of the procedure and apparatus of M. Ducamp, and it appears to us that Mr. Phillips' apparatus is not merely an imitation of that gentleman's, but is actually the thing itself. So far as we can see, there is no acknowledgment of this plagiarism, and we think it too bad that a gentleman should write a work of three hundred pages, purporting to be an original Treatise on Stricture, and passed upon the public as such, when the very marrow and essence of it, that which affects to recommend a peculiar practice, is little more than a translation. Novelty we do not at this time of day expect, but surely it is not right to adopt the inventions of another without acknowledgment, or with one so ambiguous as not to deserve the denomination. We are sorry to be compelled to make these reflections, but we should not do our duty as journalists did we not denounce such open appropriation of other men's ideas. That this is no idle accusation will, we think, be too evident from the comparison of the following descriptive passages. The first is Mr. Phillips' description of this "model sound."

"FIG. 1.—The model sound. It is formed as follows: we take a hollow bougie; through this we draw, as firmly as possible, a mesh of cotton; when it has arrived near the extremity, we cut off the cotton which has been drawn through, leaving about half an inch projecting beyond the bougie; this is dipped into a composition formed of equal parts of *bees' wax, diachylon, and shoemakers' wax,* is allowed to cool, and is then rolled between two pieces of wood or stone until it becomes in diameter nearly uniform with the other portion of the bougie." 318.

Contrast this with the description of M. Ducamp's "exploring catheter," in the Number of this Journal to which we have already alluded. It will be found at page 50. M. Ducamp observes that, to take an impression of the stricture, he uses what he calls an exploring catheter, which he then proceeds to describe.

"I have catheters of the Nos. 8, 9, and 10, open at both ends, and marked with the divisions of the foot; the anterior opening of these instruments must be about half the size of the other; I take a bit of sewing silk, and having tied several knots in it, and dipped them in melted wax, I round off the wax in the manner represented in fig. 7, plate I. By means of a bit of edging I pass the silk into the catheter, entering at the larger opening; when it has reached the other opening, the bulb formed by the knots covered with wax is detained, while the silk passes on and forms at the extremity of the catheter, a pencil of fine downy threads, both soft and strong; or else I pass the bit of flat silk through four little holes situated near the end of the instrument, and uniting them together in a knot, I afterwards spread them out in the form of a pencil. This pencil is soaked in a mixture composed of equal parts of yellow wax, diachylon, shoe-maker's wax, and resin; I take a sufficient quantity of it to enable it when rounded off to equal the bulk of the catheter. I let this *moulding wax* grow cold, and softening it between my fingers, roll it upon some hard polished surface. I cut this kind of bougie added to the gum-elastic canula, at about two lines from the extremity of the latter, and round off the wax like the end of a catheter. By this arrangement, the moulding wax, mingled with the filaments of silk, becomes incorporated with them, and cannot fall off."

It must be owned that the English model sound is a monstrously close copy of the French exploring catheter. The epithets might be reversed with some propriety, M. Ducamp having the fairest title to the model, and

Mr. Phillips to the explorer. The caustic apparatus is a copy of the same closeness, but the length of M. Ducamp's description, and the brevity of Mr. Phillips' prevent us from placing side by side this—

Counterfeit presentment of two brothers.

If, however, our readers will take the trouble to collate and compare M. Ducamp's work, or our notice of it, and Mr. Phillips', we fear they will rise from the perusal of the latter with sentiments as unfavourable to the latter gentleman's originality, as we are compelled to do.

There is one point to which we will advert a little in detail. Mr. Phillips denies that contraction follows cauterization, and on this he mainly grounds his advocacy of the cauterizing method.

"The arguments of the adversaries of the operation rest on a small number of examples of consecutive contraction, and on a supposed analogy which they seek to establish between it and other diseases. They have compared the destruction of stricture by caustic to the ablation of a part of the skin and common integuments, and the cauterized surface of the canal to a wound with loss of substance. It is by no means difficult to shew the inapplicability of this illustration :—to make the cases analogous, they must establish, that wounds of the skin, with loss of substance, and contused wounds followed by eschars, are similar to those solutions of the continuity of the canal which are consequent upon the application of caustic. But as the majority of strictures is produced by local indurations of the parietes of the canal, we may with more truth compare them to indurations developed immediately beneath or in the substance of the skin. Instead, however of comparing strictures to any other affection, we shall be more advantageously employed in watching carefully the progress of the disease and the effects of the remedy we employ. If, by affirming the existence of consecutive contraction after the skilful application of caustic, the adversaries of cauterization contend that such contraction ordinarily occurs, their deductions are, I believe, unsupported by experience ; and, as far as my observations have yet proceeded, it has not been found that the destruction of stricture by caustic, properly applied, has been succeeded by any disposition in the canal to contract anew. It is true that an extreme case may be occasionally presented ; but it would be as absurd to adopt our general treatment for extreme cases as it would be to take the exception for the rule.

In the case of stricture produced by induration, caustic destroys the morbid protuberance, which generally yields easily to its use ; and if we limit its action to the induration which projects into the interior of the canal, and do not destroy the subjacent healthy tissue, the parietes of the canal are preserved, and a healthy surface will be presented. There will be here no granulations produced, since we shall merely remove a morbid tissue ; and a thin cicatrix may cover the cauterized surface without consecutive contraction. A similar effect occurs every day in superficial burns, where the fibrous tissue has not been destroyed. After actual loss of substance, unless suppuration be really established, no fibrous tissue is generated, and no consecutive contraction occurs. The analogy on which the adversaries of cauterization have relied is not, then, exact, except in a very few strictures in which an actual loss of the healthy tissue is produced, and is succeeded by a secretion of true pus, and the development of a fibrous cicatrix. But it is stated, as an incontrovertible position, that the canal contracts by little and little after the complete destruction of ordinary strictures, and that these consecutive strictures are even much more difficult to cure than those which have not been cauterized. I am far from wishing to dispute facts asserted by authors of great respectability ; but, if we examine those cases which have been sufficiently described, we shall be convinced, from the manner in which the caustic has been applied, from the pains which have been experienced by the patient, from the (often incredible) number of applications which have been made, that false passages were formed, and that the remedy had acted upon the healthy portion and destroyed the healthy mucous membrane, probably in its entire thickness." 211.

It will be observed that Mr. Phillips denies the analogy between contraction after loss of substance, and the state of parts produced by the application of caustic. Where the mucous membrane is destroyed by the caustic there is loss of substance, and we contend that where this membrane is quite destroyed, as for instance in dysenteric ulceration of the large intestine, there is consequent contraction. But Mr. Phillips implies that in very many instances the mucous membrane is not destroyed. Now, in cases of old stricture, in ordinary cases indeed of stricture, of any degree of severity, the thickening, so far as we have examined the parts, has not been in the mucous tissue itself, but in the cellular, immediately external to it, or in the fibrous tissue external to that. If we look at contractions of other tubes lined by mucous membranes, we find the same fact, the contraction not depending on the thickening of the mucous tissue itself, but of the tissue or tissues external to it. We therefore contend that, in the great majority of cases, if caustics be applied from within outwards so as to destroy the thickening which commonly produces stricture, it can destroy it only by first destroying the mucous membrane that is placed between them.

But we doubt whether the destruction of the stricture be so simple a matter as Mr. Phillips imagines. One would think, from reading his description of the process, that it resembled very closely the planing of a piece of wood, when, the superabundant material being removed, the parts are of the desired figure and dimensions. Mr. Phillips must recollect that the caustic acts by producing an eschar, that the eschar is thrown off by inflammation, and that so long as his caustics are employed, a certain degree of inflammation must exist. Now, as chronic inflammation produced the thickening and the stricture in the first instance, there is no good reason why the inflammation excited artificially should be just what is required and no more, should be just sufficient to remove the thickening, but not adequate to perpetuate or regenerate it.

Analogies are, so far as we can understand them, against the employment of caustic. If we wish to produce contraction in a tube, we usually employ caustic—if there be an artificial opening we produce eschars, in order that the subsequent contraction may close it. The induration and contraction that follow burns are often the consequences of chronic inflammation remaining after the cicatrization of the burn, rather than of the burn itself. But we ask if any surgeon would expect to effect the reduction of that thickening by touching it every two or three days with caustic.

The conclusion then to which we are led to arrive is this, and, after all, it is more founded on facts than on reasonings. In the overpowering majority of *all* cases, in ninety-nine out of the hundred, dilatation, if practised by a dexterous person, is sufficient to enlarge the canal in an adequate manner, is attended with less pain, fewer inconveniences, and fewer risks, than any other method, and is only subject to the objection, to which we, in our consciences, believe all other methods are open also, of not proving a permanent cure. The patient must make up his mind to the passage of an instrument occasionally for many years, if not for the remainder of his life. This is an alternative to which we find patients submit very willingly, and is much more exclaimed against by those who advocate other modes of treatment, than by those who have to submit to it.

With respect to cauterization, we are confident that it is not necessary in ordinary cases; and we doubt its superior celerity or permanency of good, and its equal safety, in the greater number of severe cases. By the method of applying it from within outwards, it is first necessary to get an instrument into or through the stricture, and if that be effected, the worst of the difficulty is vanquished.

Experience, however, is the great test of opposing modes of treatment. Mr. Phillips says experience is in his favour, and brings forward cases to support him. We know not how it is; but it always happens that, whatever be the drug or the operation vaunted, there is never a lack of facts in its favour. We will venture to affirm that Goss and Co. have as many cases, and apt ones, too, as would be necessary to establish any orthodoxy or heterodoxy whatever. Nay, we will allow, and we do it in perfect sincerity, that these facts are not false ones. The majority of cases do well under opposite methods of treatment, provided that they be directed with moderate judgment. There is no difficulty, then, in procuring cases, which, to the public, may appear satisfactory evidence, but to those who have much experience, or much caution, have by no means so imposing a character. We venture, then, to say, in reference to the present subject, that we believe that the experience of surgeons in the best practice in this country is in favour of dilatation. We do not bring forward their evidence *against* Mr. Phillips' or M. Ducamp's method of cauterization, because it will be said that the latter has not been sufficiently tried. We admit most fully and freely the validity of this objection. But we do bring forward their experience, and we do it with the utmost confidence, *in favour* of dilatation; we protest, in their name, against the character which the advocates of cauterization give it; and as their advocacy of the latter is mainly founded on their depreciation of the former, it follows of course that if that depreciation be exorbitant, the grounds for their recommendation of caustic are, *pro tanto*, fallacious.

Whilst we have thus expressed our opinions on this subject, we by no means wish to preclude investigation or experiment. Knowledge should not, cannot be stationary, and all disputes of this description are calculated to elicit truth, and promote the ends of science. But we find fault with Mr. Phillips for not acknowledging more openly and more explicitly the amount which he owes to M. Ducamp; and if a new practice is to be advocated or introduced, let its true owner, at all events, have the merit of it.

Mr. Phillips makes a few remarks upon incision, and with these we must close this notice, already too extended. He objects to Mr. Stafford's instrument, because he considers its employment attended with risk. We share his sentiments on this occasion, and we must say that we think the "lancetted stilette" either unnecessary or dangerous. We are sure that the more dexterous a surgeon is, the fewer will be the occasions on which he will cry out, like weak politicians, for force. The punctures of the bladder, and caustics, and cuttings are, in too many instances, but a clumsy apology for more clumsy manipulation. Mr. Phillips, however, has an instrument of his own, although he condemns Mr. Stafford's. The following is a brief account of it.

"In those cases where the stricture is of a valvular kind, where it retreats before the bougie, and where, from this circumstance and its trifling extent, some uncertainty would attend any attempt to cauterize it, the cutting instrument I have invented may be employed with the most complete success. Where the induration has become so excessive that the indurated matter acquires almost a horny texture, and where the extent of surface which it occupies is inconsiderable, and, lastly, when any circumstance renders it necessary that the stricture should be very rapidly destroyed, in all these cases I do not hesitate in recommending the employment of this instrument. By the operation which I have introduced, the obstacle is instantly removed, after which an elastic catheter is passed into the bladder, for the purpose of protecting the incised portion of the canal from the irritation which would be produced, by the escape of the urine, during the evacuation of the contents of the bladder. The instrument which I have constructed for the purpose is described in the Appendix, where a representation thereof is given. It presents a circular cutting edge, is introduced into the urethra in a canula, and when the canula is in contact with the stricture, a probe or stilet, situated in the centre of the cutting portion, is gently introduced into the orifice of the stricture, and serves to maintain the instrument in the proper position in the canal. The cutting instrument is then advanced, placed in contact with and pressed against the stricture, a circular motion being at the same time given to it, similar to that given to a trephine, and in two or three moments the stricture is removed and the canal free. The operation may be performed with much facility, is wholly unattended by danger, and the pain is not much more considerable than that which accompanies the application of caustic." 222.

Mr. Phillips speaks doubtingly of the employment of incision, and remarks that he "cannot conceal from himself the conviction, that much dexterity and long experience in the use of the instrument are necessary." We suspect that, with much dexterity and long experience, neither this nor any other cutting instrument will be found necessary.

We would wish to state most explicitly, that we do not condemn indiscriminately caustic or incision; on the contrary, there are cases in which either may be advantageous. But we do believe that those cases are the exceptions, not the rule; and it is of making either the staple treatment of stricture that we altogether disapprove. What the cases are to which caustic or incision is applicable, it is not for us at present to enquire. In conclusion, we beg to observe that in the criticisms we have employed, we have intended nothing personally offensive to Mr. Phillips. We have taken the liberty of examining his doctrines, and the judicious part of the public must arbitrate between us.

